# The MFM program: a successful model in the field of food addiction recovery

**DOI:** 10.3389/fpubh.2025.1630084

**Published:** 2025-08-26

**Authors:** Esther Helga Gudmundsdottir, Veronique Rynn

**Affiliations:** ^1^INFACT School, Reykjavik, Iceland; ^2^Department of Psychology, Addiction Psychology Program, Arizona State University, Tempe, AZ, United States

**Keywords:** food addiction, abstinence, recovery, 12-step facilitation, ultra-processed food

## Abstract

This article outlines how one program in Iceland, the MFM program, is helping clients recover from food addiction and ultra-processed food addiction. The MFM program, founded and developed by Esther Helga Gudmundsdottir (EG), arose from her own recovery from food addiction. Seeing the gap in services for clients with food addiction and ultra-processed food addiction, EG set out to develop a treatment plan based on everything she learned over the years and through her experience and trainings.

## Background

The term “food addiction” was first introduced in the medical literature in 1956 by Theron Randolph (1). Since then, the concept of food addiction has gained greater understanding and acceptance. Since 2006, the number of studies on food addiction have increased dramatically, indicating a greater awareness and acceptance of this disease (2). The term “ultra-processed food addiction” has also gained greater acceptance in recent years as correlations are being found between obesity and increased ultra-processed food (UPF) consumption (3). The MFM (Medferdar go Fraedslumistod vegna Matarfikuar in Icelandic - translates to *Educational Center for Food Addiction Counseling and Treatment*), an outpatient center for food addiction assessment, counseling and treatment in Reykjavik, Iceland, was founded by EG. Having recovered from food addiction, she saw a gap in assessment and recovery in her country. She felt called to fill this gap by contributing to education and treatment for this disease as there was currently very little awareness and understanding that sugar and certain foods can cause a substance use disorder - with the substance being food.

There were two 12-step programs for compulsive eating in Iceland at the time: GreySheeters Anonymous (GSA), which identifies a need to abstain from grains, sugars and flour and Overeaters Anonymous (OA), where a client identifies and chooses an eating plan. As this article will describe, The MFM program provides greater professional assessment, education and ongoing support for clients. Prior to founding the MFM program, EG began a process of addiction studies, garnering diplomas as an addiction counselor, then training in clinical supervision. She then undertook the three-year, experimental program for food addiction professional at ACORN (now SHiFT, located in Florida and Vancouver). When EG first opened the MFM center, it quickly progressed from one-on-one sessions to weekend recovery days, daily support and aftercare groups. The MFM program follows the ASAM guidelines for addiction treatment as well as incorporates the latest developments in the burgeoning field of food addiction (2024) (10).

## Case presentation

**Figure 1:**
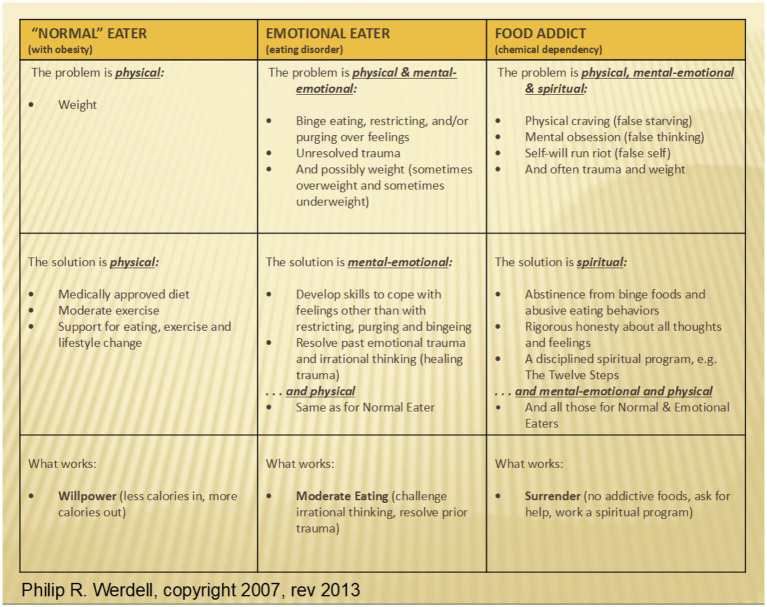
Classification of eating style and corresponding interventions (11).

In this section, details of the MFM program will be discussed. In the MFM Program, clients seeking assistance with their food addiction receive instruction and courses that allow them to explore the addictive model of treating food addiction. Prior to entering the program, clients are screened and an assessment interview is completed with a staff member. One of the assessments used in this process is the Yale Food Addiction Scale 2.0 Assessment developed by Ashley Gearheardt and her team while at Yale (4). This is where the concept of food addiction is explained and explored, personally, with each client. From this, each client is assessed for appropriateness for the MFM program. Key topics discussed during this initial intake and assessment are the differences between the diseases of obesity, the disease of eating disorders and the disease of chemical dependency on food, or food addiction (see Figure 1).

**Figure 2:**
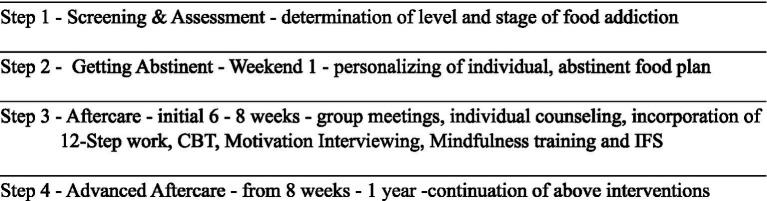
Breakdown of program.

**Figure 3:**
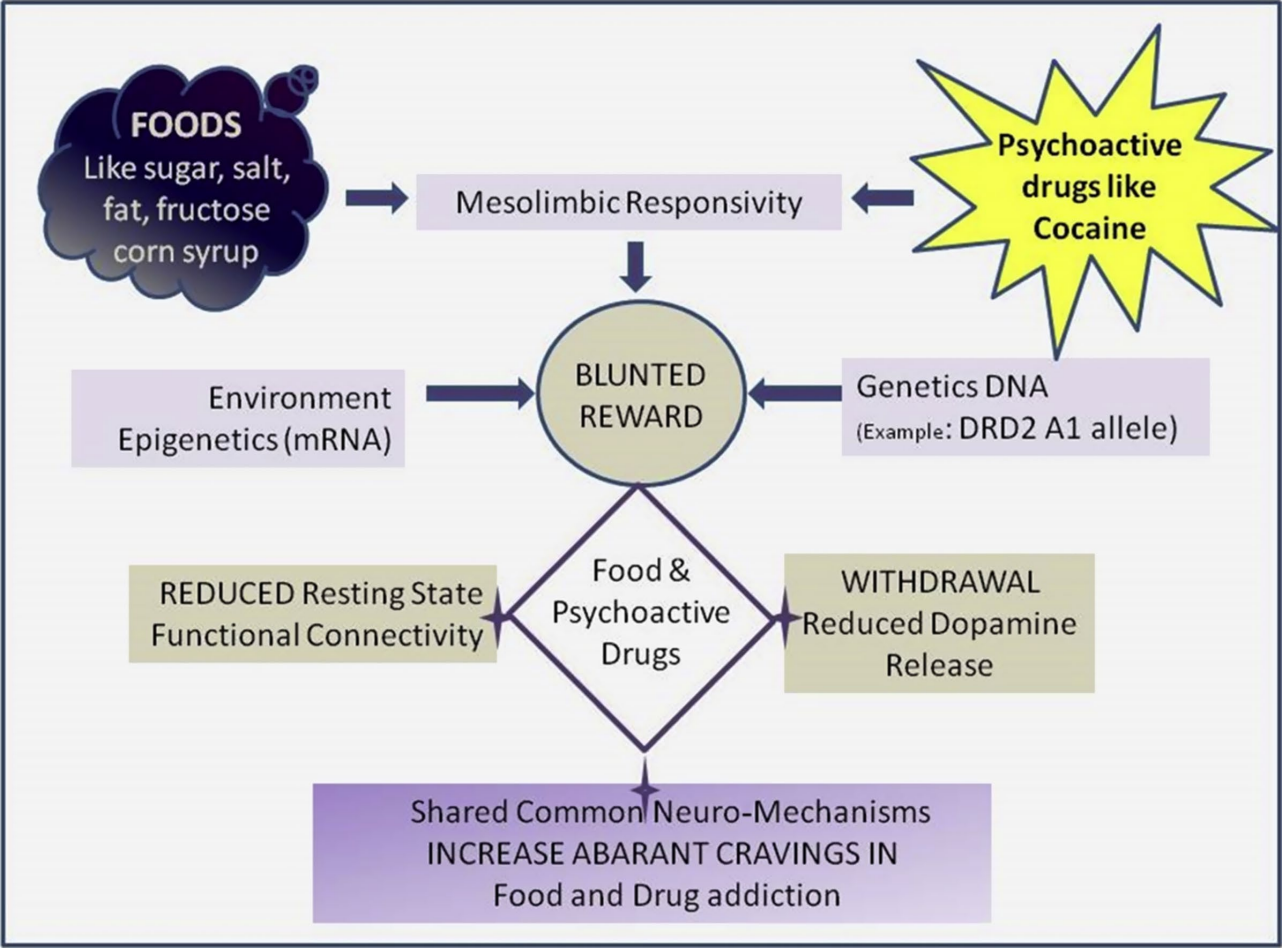
Similarities of pathway activations of food and substance use. From a shared molecular and genetic basis for food and drug addiction (9).

The MFM program (see Figure 2) begins with a weekend where clients explore an abstinent food plan. Abstinence is the non-consumption of the following addictive food substances: flour, sugar and processed foods. An abstinent food plan centers on eliminating sugar, flour, UPFs and other trigger foods. It also includes a structured timeline of eating at set periods during the day, excluding snacks and consuming a weighted and measured amount of food at each meal. Trigger foods are those foods that are determined to be difficult to stop eating once eating is initiated, are reached for when client is undergoing an emotional, physical or spiritual crisis, or keep the client in an uncentered state. These trigger foods overwhelmingly contain sugar, flour and UPFs. Clients learn that just as other substances can become addictive - heroin, cocaine, alcohol and others - so can foods containing sugar and other highly processed substances. It has been shown that certain foods excite the same neural pathways as these non-food substances (5, 6, 12) (see Figure 3). The addictive component of some food substances has been well studied and documented.

The first day of this weekend involves complete abstinence from their trigger foods with the support for the withdrawals that occur. With this initial detox, additional trigger foods are potentially identified and a plan of eating is provided that detoxes their body from whatever is keeping their physical body in the addictive cycle. Cooking classes are also offered during this first weekend to help clients explore the many food options they have while leaving the trigger foods behind.

Clients must abstain from food substances that may have been part of their favorite recipes and meals but they also learn of the breadth of choice available to them. Toward the end of the first morning, the menu for lunch is explained and participants observe the cook’s final preparations. These preparations include simple combinations of seasoning and cooking methods that add flavor to food. Clients then sample the abstinent food that was just prepared.

On the afternoon of the second day, questions are entertained about the meal, materials covered in the morning and anything else clients are concerned about. Clients are introduced to food addiction as a brain disease and the inevitability of needing to deal with difficult feelings, irrational thinking and disturbances of the will. This psychosocial portion of the program is an ongoing one that is at the root of food addiction recovery. One aspect of this psychosocial learning relates to accountability, which is demonstrated with the submission of daily check-ins with mentors in the areas of physical, mental and emotional health. Through these daily check-ins, clients can refine their agency with actions that promote and support their recovery.

The focus of the latter part of this first day includes assisting clients with refining what each client needs in order to go home and prepare abstinent meals for themselves and, oftentimes, their families. The following issues are addressed: time management, food shopping, how to manage family meals if one person is eating differently and others issues that arise when one person makes changes within a family system. After these issues are explored, clients go home to start their new way of eating and are asked to notice problems or difficulties they may encounter along the way.

On Sunday of the first weekend, a check-in occurs to see how home abstinent meal preparation went. The discussion ranges from practical problems to processing the emotions that surface during this process. Clients are encouraged to “see themselves as someone who struggles with an addictive substance” for the next several weeks or months, with the idea being to treat food addiction like what it is - a true addiction like any other. After learning about addiction as a brain disease early in the MFM program, this is something that clients are capable of doing and one that can reinforce their recovery. Clients are required to call an MFM support person daily to report the food they are going to eat the next day, along with a self-assessment of their emotions, issues they struggled with during the day, spiritual questions that arise, and where they feel they are needing help. Relapse, a common occurrence when working with any addictive substance, is also addressed. Questions about the client’s recovery are explored to help the support person assess what is most needed for that individual’s recovery.

After this intensive weekend, clients attend a weekly 2-h group meeting, where participants discuss problems they encounter in their new way of eating. As clients settle into this new way of abstinent eating, the following topics are addressed in the form of lectures:

- What is the scientific basis of food addiction and food addiction treatment?
- What resources are available for clients in the community, such as 12-steps, GreySheeters Anonymous (GSA) or Overeaters Anonymous (OA)?
- How can one stay “food addiction abstinent” at work, at social events or when traveling?
- What is meant by food addiction as a spiritual disease?
- What questions or problems does eating abstinently bring up in individuals in the areas of body, mind, spirit?

Once an abstinent plan is firmly in place for clients, participants are given Step 1 assignments. Writings are read in group and discussed. Clients are encouraged to continue to explore, concurrently, step topics in their local communities in OA and GSA meetings. Many clients choose to continue with the MFM program for their support and enroll in a subsequent 10 week session. Some clients choose to transition from the MFM program to local 12-step support groups and others choose to continue their recovery on their own. The most popular option for the majority of clients continues to be signing up with the MFM program for another 10 weeks.

In subsequent 10-week sessions, more rigorous 12-step work is explored, with increasing understanding and acceptance of the need for powerlessness when struggling with addictive substances in this case, food. Twelve Step Facilitation (TSF) forms the foundation of clients’ continued work in the MFM program. This model is aligned with the findings of a gold-standard Cochrane Review by Kelly et al. (7), which demonstrated that TSF is at least as effective as cognitive behavioral therapy (CBT) in promoting long-term abstinence and engagement in recovery among individuals with substance use disorders. By embedding these evidence-based principles into the programme, MFM offers a structured and clinically credible approach to treating food addiction as a substance use disorder.

By this point, clients are understanding that merely being abstinent for a short period of time does not immediately heal the brain - it is also necessary to continue embracing powerlessness to more deeply heal the brain, a cornerstone of TSF. Deeper emotional issues are addressed and some clients decide to add individual therapy to the MFM program’s group work. As has been observed, clients start to feel more and more comfortable with attending 12-step meetings and often embark on this work, on a daily basis, after choosing a sponsor to work with them on this journey.

As with any program that works with clients struggling with substance use disorder, the MFM program is focused on client-centered care and providing the most personalized experience possible for the client. The more personalized, the greater the clients’ success. Another aspect that is a product of TSF is the client’s examination of what spirituality means to them, individually. Puchalski et al. (8) defines spirituality as:

“Spirituality is a dynamic and intrinsic aspect of humanity through which persons seek ultimate meaning, purpose and transcendence, and experience relationship to self, family, others, community, society, nature and the significant or sacred. Spirituality is expressed through beliefs, values, traditions, and practices.”

While there was no operating definition of spirituality offered to the cohort for purposes of the survey, future research would benefit from a definition and a means to quantify growth and progress in this domain (12, 13).

## Methods

In this retrospective study, web-based questionnaires were disseminated to 513 clients, 325 clients opened the link and 228 clients answered. These clients were from the MFM’s database over a 9-year period. The questionnaire for this case study, administered in 2017, was a revised version of one used by ACORN. Dr. Olof Asta Olofdottir, the Nursing Department Head at Iceland University, oversaw the collection of data. This cohort’s length of treatment in the MFM program ranged from 3 months to greater than 12 months. Ethical approvals were received by the National Bioethics Committee (VSN) and the Data Protection Authority of Iceland.

### Participants

87% were women between the age of 30–60, with 60% of them with an academic degree, with most reporting an eating and weight problem from early age (Tables 1, 2). Binge eating had been experienced by 94% of participants with purging and vomiting experienced by 20%. Dieting had predominantly started in childhood, from the ages of 5–15 and again from ages 20–30, usually correlating with pregnancy. Fasting and/or starving behaviors was experienced by 65% of subjects and over-exercising was experienced by 20% of this cohort. In addition to their current food addiction issues, other psychosocial elements existed - 42% experienced other types of addiction, 68% experienced abuse and trauma and 50% disclosed depression and anxiety.

**Table 1:** Description of participants.

Gender	Women 87%, Men 13%
Age	30–60 years of age
Education	60% had academic degrees
Prior attempts	90% sought medical and fitness solutions to weightless

**Table 2:** Food addictive behavior of participants prior to entering MFM program.

Binge Eating - 94%
Binge Eating periods from 2 times/day to 8 times/wee (80%)
Purging and vomiting - 20%, with 30% had binged on food in the past 24 h
Fasting and/or starving (65%, from 1 month - 1 year)
Over-exercising (20%)
Used medication for weight loss (20%)
70% have used from 7 to 10 ways to diet and lose weigh

## Results

Of the 513 questionnaires mailed, 228 questionnaires were answered (a 44% response rate) (see Table 3). Data collected showed 30 percent of clients who completed the MFM weekend were abstinent from sugar and grains (along with other trigger foods) and from day 1 had been maintaining healthy weight loss without obsessive thinking about food, a hallmark of decreased cravings. One third had some slips and false starts, along with periods of relapse but ultimately were abstinent at the end of the questionnaire. The remaining 40% were not abstinent at the time of the survey but many were still in the MFM program or in 12-step programs, stating they felt they were making progress.

**Table 3:** Topics addressed in questionnaire.

Pre-Recovery	Recovery
Age	What foods were they abstinent from
Gender	Participation in 12-step process
Education, income	How they felt about the MFM services
Life events	
Previous weight loss methods	
Co-occurring disorders (i.e., mental health issues)	
Addictions/cravings	
How cravings affected health on a holistic level	

### Longer terms findings

Additional data collected showed that over 60% of participants had abstinent periods from greater than 1 month up to several years. 37% reported stable abstinence of greater than 1 year, with weight loss and other positive emotional physical outcomes. 20% of participants stopped treating themselves like food addicts immediately after leaving the program. Most of the MFM alumni group actively continued their participation in 12-step support groups. They indicated a higher positive view of these support groups than before they started the MFM program. Over 70% reported abstinence greater than 1 month, along with strong positive emotional growth. The length of abstinence varied from 1 to 6 months (30% of participants) to greater than 1 year (30%). 90% of the respondents lost weight - 50% lost 25–60 pounds and 17% lost more than 60 pounds.

### Recovery actions of alumni

30% reported using the structured food plan provided by MFM and were still weighing and measuring their food at the time of the questionnaire; 30% reported a practice of prayer and/or meditation; 40% reported using some other 12-step tools such as attending GSA and OA meetings; 23% considered themselves active members of a 12-step program and 10% were actively sponsoring others with similar issues with food addiction.

### Overall experience of the program

The MFM program proved to be effective (rated a 4 out of 5 on a scale of 0–5) by 65–75% of respondents. Reports included having greater capacity to view life in a positive manner and developing increased spiritual strength. 10% reported either neutral or negative views of the program. 30% did not answer the question asking about current abstinence. One possible interpretation is that those struggling with abstinence were still in denial about food addiction being a disease and perhaps felt guilt or shame in not being able to answer affirmatively. In addition to the above, there was also an open-ended question asking about their overall experience. 53 participants wrote narratives about their recovery, including their difficulties and experiences with relapse.

## Discussion

This cohort demonstrated the maintenance of weight loss and relief from secondary medical problems paralleling results from ACORN and Glenbeigh programs. The implications arising from the data collected suggests many potential avenues for further exploration of models of care in the field of food addiction. In Iceland, the MFM center is the only provider of screening, assessment, and treatment for food addiction. People pay for MFM services out-of-pocket, which is the current state of all such programs around the world. Collecting data that suggests good outcomes for clients struggling with food addiction will go a long way toward contributing to the process of the APA’s DSM-5 and the WHO’s ICD-11’s inclusion of food addiction and UPF addiction into their list of reimbursable conditions. The prevalence of conditions associated with obesity — cardiovascular disease, diabetes, autoimmune diseases — can be stemmed if more public health attention is directed at food addiction assessment and treatment.

Some limitations of this study include the lack of a control group, the absence of a method to quantify clients’ spiritual growth and the retrospective design. Self-selection bias is likely a factor in surveys responses.

As other options such as bariatric surgeries and the GLP-1 medication become mainstream, people who get assessed with food addiction or UPF addiction as a substance use disorder may still need treatment for food addiction. Data shows persons taking the GLP-1 medications do not stay on them long-term, and food addiction treatment programs, such as the MFM program or a replicable model, would provide a solution for those unable to stay on the medications.

Future exploration in the field of food addiction treatment could include a spirituality scale to quantify the growth and transformation attested to by participants.

## Author’s note

ACORN (now SHiFT) - an intensive residential treatment program for food addiction. ACORN/SHiFT is adapted from the Minnesota Model of Chemical Dependency to incorporate food as the dependent substance. Glenbeigh Psychiatric Hospital was an inpatient facility that treated food addiction. Programs like the Glenbeigh Psychiatric Hospital in Tampa (no longer in existence) and ACORN (now known as SHiFT) paved the way for the treatment model at MFM. Additionally, the information and results provided in this study were presented by Dr. Olof Asta Olafsdottir at an International Conference on Obesity in 2017.

## Data Availability

The raw data supporting the conclusions of this article will be made available by the authors, without undue reservation.

## References

[1] Randolph TG. The descriptive features of food addiction: addictive eating and drinking. Q J Stud Alcohol. (1956) 17:198–224. doi: 10.15288/qjsa.1956.17.198, PMID: 13336254

[2] Meule A. Back by popular demand: a narrative review on the history of food addiction research. Yale J Biol Med. (2015) 88:295–302. PMID: 26339213 PMC4553650

[3] LaFata EM Allison KC Audrain-McGovern J Forman EM. Ultra-processed food addiction: a research update. Curr Obes Rep. (2024) 13:214–23. doi: 10.1007/s13679-024-00569-w, PMID: 38760652 PMC11150183

[4] Gearhardt AN Corbin WR Brownell KD. Development of the Yale food addiction scale version 2.0. Psychol Addictive Behavs. (2016) 30:113–21. doi: 10.1037/adb0000136, PMID: 26866783

[5] Schienle A Schäfer A Hermann A Vaitl D. Binge-eating disorder: reward sensitivity and brain activation to images of food. Biol Psychiatry. (2009) 65:654–61. doi: 10.1016/j.biopsych.2008.09.028, PMID: 18996508

[6] Srivastava A. B. Gold M. S. When food is an addiction. In: Danovitch I. Mooney L. J. (Eds.). The assessment and treatment of addiction: Best practices and new frontiers. Elsevier. (2019) 197–205. doi: 10.1016/B978-0-323-54856-4.00014-6

[7] Kelly JK Humphreys K Ferri M. Alcoholics anonymous and other 12-step for alcohol use disorder. Cochrane Database Syst Rev. (2020) 3. doi: 10.1002/14651858.CD012880.pub2, PMID: 32159228 PMC7065341

[8] Puchalski CM Vittilo R Hull SK Roller N. Improving the spiritual dimension of whole person care: reaching nation and international consensus. J Palliat Med. (2014) 17:642–56. doi: 10.1089/jpm.2014.9427, PMID: 24842136 PMC4038982

[9] Gold M Badgaiyan R Blum F. A shared molecular and genetic basis for food and drug addiction. Psychiatr Clin. (2015) 38:419–62. doi: 10.1016/j.psc.2015.05.011, PMID: 26300032

[10] Clinical Guidelines Committee (CGC) Members, ASAM Team, AAAP Team, & IRETA Team. The ASAM/AAAP clinical practice guideline on the Management of Stimulant use Disorder. J Addict Med. (2024) 18:1–56. doi: 10.1097/ADM.0000000000001299, PMID: 38669101 PMC11105801

[11] Foushi M Weldon C Wendell P. Food addiction recovery - a new model of professional support: The ACORN intensive. Sarasota, Florida: Evergreen Publications (2007). 70 p.

[12] Schulte EM Avena NM Gearhardt AN. Which foods may be addictive? The roles of processing, fat content and glycemic load. PLosone. (2015) 10:E117959. doi: 10.1371/journal.pone.0117959, PMID: 25692302 PMC4334652

[13] De Brito Sena MA Damiano RF Lucchetti G Peres MFF. mDefining spirituality in healthcare: a systematic review and conceptual framework. Front Psychol. (2021) 12. doi: 10.3389/fpsyg.2021.756080, PMID: 34867654 PMC8637184

